# C_60_ in a peptidic cage: a case of symmetry mismatch studied by crystallography and solid-state NMR

**DOI:** 10.1107/S2052520620009944

**Published:** 2020-08-29

**Authors:** Miroslaw Gilski, Piotr Bernatowicz, Arkadiusz Sakowicz, Marek P. Szymański, Aldona Zalewska, Agnieszka Szumna, Mariusz Jaskólski

**Affiliations:** a Department of Crystallography, Faculty of Chemistry, Adam Mickiewicz University, Poznan, 61-614, Poland; b Center for Biocrystallographic Research, Institute of Bioorganic Chemistry, Polish Academy of Sciences, Poznan, 61-704, Poland; c Institute of Physical Chemistry, Polish Academy of Sciences, Warsaw, 01-224, Poland; d Institute of Organic Chemistry, Polish Academy of Sciences, Warsaw, 01-224, Poland; e Department of Chemistry, Warsaw University of Technology, Warsaw, 00-664, Poland

**Keywords:** encapsulation, supramolecular chemistry, NMR relaxation, peptide-fullerene interactions

## Abstract

A supramolecular complex, formed by encapsulation of C_60_ fullerene in a molecular container built from two resorcin[4]arene rims zipped together by peptidic arms hydrogen bonded into a cylindrical β-sheet, was studied by X-ray crystallography, solid-state and solution NMR, EPR spectroscopy and differential scanning calorimetry (DSC). The crystal structure, determined at 100 K, reveals that the complex occupies 422 site symmetry, which is compatible with the molecular symmetry of the container but not of the fullerene molecule, which has only 222 symmetry. The additional crystallographic symmetry leads to a complicated but discrete dis­order, which could be resolved and modelled using advanced features of the existing refinement software. Solid-state NMR measurements at 184–333 K indicate that the thermal motion of C_60_ in this temperature range is fast but has different activation energies at different temperatures, which was attributed to a phase transition, which was confirmed by DSC. Intriguingly, the activation energy for reorientations of C_60_ in the solid state is very similar for the free and encaged molecules. Also, the rotational diffusion coefficients seem to be very similar or even slightly higher for the encaged fullerene compared to the free molecule. We also found that chemical shift anisotropy (CSA) is not the main relaxation mechanism for the ^13^C spins of C_60_ in the studied complex.

## Abstract 2

A molecular container with C_60_ cargo was crystallized and studied by X-ray diffraction revealing a complicated dis­order of the ligand caused by the incompatibility with its site symmetry. The tumbling of the C_60_ cargo was studied by solid-state NMR which suggested possible phase transitions, positively verified at high temperature by DSC.

## 1. Introduction   

An inherent feature of molecular capsules is the presence of a cavity that can host and sequester other molecules (guests) from the external environment. By virtue of this, molecular capsules find application in the storage of unstable and reactive molecules, and their controlled release (Mal *et al.*, 2009 ▸), as catalytic nanovessels that provide a constrictive environment for single reactions (Zhang & Tiefenbacher, 2015 ▸; Zhang *et al.*, 2017 ▸) and domino-type processes (Salles *et al.*, 2013 ▸). Interactions between a capsule and its cargo are of particular interest to molecular engineers because these are the very factors that control the thermodynamics and kinetics of the encapsulation process. However, precise characterization of the mode of such interactions is in most cases troublesome, especially for spherical guests residing in spherical cavities. Firstly, because the occupation factor (percentage of the internal volume of the cavity that is occupied by the guest molecule) is often low. For non-polar guests, an optimal occupation factor is claimed to be 55%, which is similar to the occupation factor of a non-polar liquid phase (Mecozzi & Rebek, 1998 ▸). For a polar guest, the occupation factor can reach higher values, but it is still similar to the occupation factors of polar liquid phases (*e.g.* ∼75%, as for liquid water) (Szumna, 2009 ▸). These low occupation factors mean that the guest molecule in the cavity behaves in a solution-like manner – it is thermally randomly dis­ordered. Another possible complication comes from the high symmetry of such systems, which often results in the distribution of the electron density over several symmetry-related positions. The importance of symmetry matching of the host and guest molecules should be noted, as it maximizes the number of points of contact and the efficiency of the interaction (Atwood *et al.*, 1999 ▸). All these factors, together with the typically low resolution of the diffraction data, render the precise determination of the position of guests inside cavities problematic. In this article, we report the challenging and tedious but ultimately successful path toward the resolution of the dis­ordered positions of the C_60_ spherical guest inside the cavity of a peptidic molecular capsule in a supramolecular crystal, additionally complicated by a symmetry mismatch between the host and guest components. Moreover, by measuring the solid-state ^13^C nuclear spin relaxation for the encapsulated fullerene molecule, we were able to analyze the dynamic behaviour of the entrapped fullerene over a broad temperature range. Yanagisawa *et al.* (2007 ▸) previously performed investigations of a similar inclusion complex by X-ray and NMR lineshape analysis, but the exploited model, *i.e.* an iridium porphyrin cyclic dimer with C_60_ inside the cavity, revealed no dis­order in the solid state. The ^13^C signal broadening which was noticed in the liquid-state NMR spectra indicated very likely some dynamical behaviour of C_60_, but in fact no convincing arguments for the origin of the broadening were shown.

**Figure 1:**
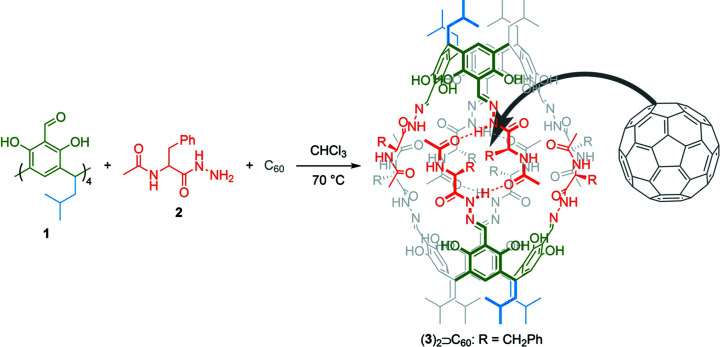
Synthesis and schematic structure of complex (**3**)_2_ 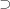
C_60_ with highlighted molecular components (blue is the lower rim, green the resorcinarene moiety, red the upper rim and gray fullerene).

Our motivation for undertaking an in-depth analysis of the interactions of encapsulated C_60_ with its host, the peptidic capsule (**3**)_2_ (Fig. 1 ▸), was stimulated by the unusually high affinity of fullerenes towards such capsules that is not intuitive in terms of intermolecular forces, because it involves interactions of C_60_ with the backbones of the peptides, while in the previously reported crystal structures of C_60_ with proteins (Kim *et al.*, 2016 ▸), more intuitive interactions involving π–π stacking with tyrosine (Tyr) side chains were demonstrated. We have recently prepared a series of cavitands similar to **3** that are composed of resorcin[4]arenes decorated with various peptidic arms (Szymański *et al.*, 2016 ▸, 2018 ▸; Grajda *et al.*, 2017 ▸; Eichstaedt *et al.*, 2019 ▸). These cavitands have vase shapes with the resorcin[4]arene parts constituting rigid bases, each equipped with four flexible oligopeptide arms. The cavitands dimerize to form non-covalent molecular capsules with an internal volume of *ca* 700 Å^3^. Interactions between the two cavitands involve hydrogen bonds between the peptide backbones zipped together by a circular pattern of hydrogen bonds that mimic the association motif of a protein β-barrel. The capsules exhibit a marked preference towards encapsulation of fullerenes (C_60_ or C_70_), both in polar (methanol, H_2_O; Grajda *et al.*, 2017 ▸; Eichstaedt *et al.*, 2019 ▸) and non-polar (CHCl_3_; Szymański *et al.*, 2016 ▸, 2018 ▸; Grajda *et al.*, 2017 ▸) environments. Such a complexation preference is quite striking since the internal surface of the cavity is largely composed of the peptide backbones, with a considerably smaller contribution from the aromatic rings of the cavitand. Moreover, insensitivity to the complexation environment is also unprecedented, because in solvents of such drastically different polarity [∊(CHCl_3_) = 4.8 and ∊(H_2_O) = 80.1] different non-covalent interactions should dominate, which in turn should lead to the formation of different complexes. Specifically, hydro­phobic effects should dominate in water, while electrostatic-based interactions should predominate in a non-polar environment. It is very unusual for these interactions, which are orthogonal under given conditions, to lead to the same type of complex. Motivated by these intriguing observations, we aimed at crystallographic and NMR investigations of the origins of the **3**/C_60_ interactions.

## 2. Materials and methods   

### 2.1. Sample preparation   

Tetra­formyl­resorcin[4]arene, **1**, and *N*-acetyl-l-phenyl­alanine hydrazide, **2**, were obtained according to literature procedures (Szymański *et al.*, 2016 ▸; Grajda *et al.*, 2013 ▸). **1** (0.125 mmol, 103 mg), **2** (0.5 mmol, 111 mg) and fullerene C_60_ (62.5 µmol, 47 mg) were dissolved in CHCl_3_ (5 ml) in a sealed pressure vial. The reaction mixture was stirred at 70°C for 4 d. The mixture was cooled and filtered. The solution was then evaporated under reduced pressure. The products were analyzed by NMR spectroscopy. The quantitative formation of the (**3**)_2_ 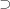
C_60_ complex was detected.

### 2.2. Crystallization   

Complex (**3**)_2_ 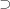
C_60_ (10 mg) was dissolved in a 3:1 (*v*/*v*) mixture (2 ml) of CHCl_3_ and EtOH, and was allowed to crystallize by slow evaporation.

### 2.3. X-ray data collection   

High-resolution X-ray diffraction data were collected at the EMBL beamline P13 of the Petra III storage ring (DESY, Hamburg, Germany) at a wavelength of 0.8266 Å, using a PILATUS 6M detector. The data were collected at the cryogenic temperature of 100 K. The diffraction data were indexed, integrated and scaled using the *XDS* package (Kabsch, 2010 ▸). Scaling of the intensities was acceptable and similar in the space groups *I*4 and *I*422, but for reasons explained below, we first chose the former. The correct latter space group was used for the final model refinement. The X-ray data collection statistics are summarized in Table 1 ▸. [Raw diffraction images for the (**3**)_2_ 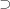
C_60_ crystal were deposited in the RepOD repository at the ICM of the University of Warsaw and are available for download with the following digital object identifier (DOI): http://dx.doi.org/10.18150/XMEUNL.]

### 2.4. Structure solution and refinement   

The crystal structure of (**3**)_2_ 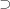
C_60_ was solved in the space group *I*4 by direct methods using the *SHELXT* program (Sheldrick, 2015*a* ▸). The preliminary model of the structure generated by *SHELXT* included two quarters of a single resorcin[4]arene skeleton decorated with l-phenyl­alanine arms and lower-rim alkyl chains (*i.e.* a quarter of the entire resorcinarene–peptide capsule) arranged around a fourfold axis, plus a few randomly placed fullerene atoms.

**Figure 2:**
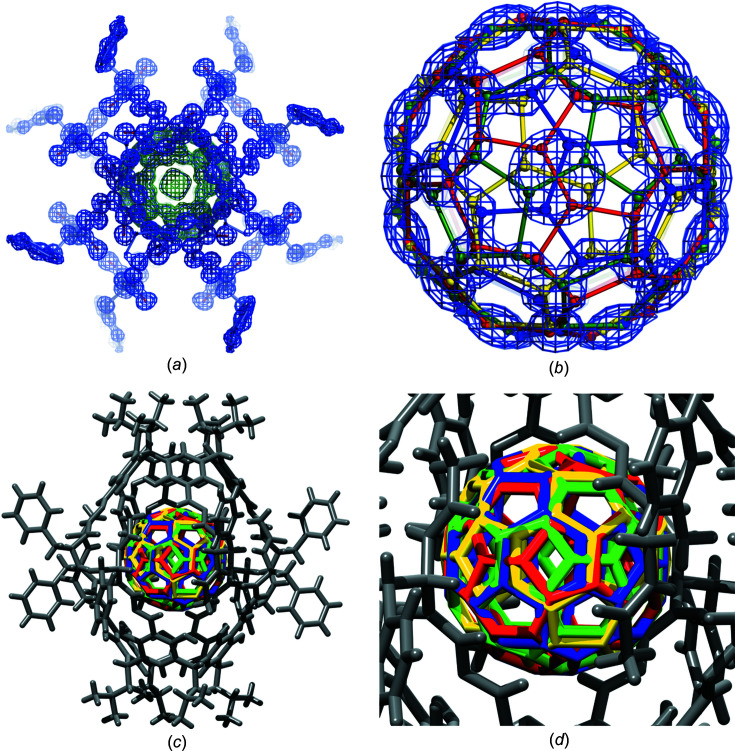
X-ray crystal structure of (**3**)_2_ 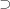
C_60_. (*a*) Initial *mFo*–*DFc* electron-density OMIT map (green) contoured at 3.0σ of the fullerene molecule and the 2*mFo*–*DFc* map (blue) of the whole capsule contoured at 1.5σ (the asymmetric unit contains only 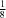 of the capsule, *R* ≈ 30%). (*b*) Eight halves of the fullerene moiety in the unit cell (forming four molecules of C_60_) shown in the final 2*mFo*–*DFc* electron-density map contoured at 2.0σ. The four complete dis­ordered C_60_ molecules (two pairs: red–green and blue–yellow) lying on the fourfold axis are shown, viewed along the crystallographic *c* axis. (*c*)/(*d*) A stick representation of the final structure with refined dis­order of the C_60_ molecule.

After a few cycles of isotropic refinement with *SHELXL* (Sheldrick, 2015*b* ▸), which converged at *R* ∼24%, the initial electron-density maps were generated and were thoroughly inspected with the *COOT* program (Emsley *et al.*, 2010 ▸). The maps clearly showed well-defined positions for all atoms forming the capsule and a characteristic spherical density corresponding to the fullerene molecule. However, the number and distribution of the peaks at the fullerene site indicated a massive dis­order of this molecule. Before the next round of refinement, the model generated by *SHELXT* was revised manually by assigning proper atom types and removing all spurious fullerene atoms. After a round of least-squares refinement of this model, which corresponded to about one half of the expected asymmetric unit (ASU) content in the space group *I*4, the *R* factor increased to ∼30%, but it turned out that the electron density of the fullerene molecule was still clearly visible with pronounced, fairly well separated, peaks (Fig. 2 ▸ *a*). Interpretation of this map was, however, very difficult because we were dealing with the effect of overlapping partially occupied fullerene molecules. In addition, the fullerene molecule was located on the fourfold axis (passing through two opposite C—C bonds, each shared by two hexagons), which is not the site symmetry of the C_60_ molecule.

The location of the electron density corresponding to the C_60_ molecule within the unit cell, the *I*4 symmetry and the arrangement of the peaks at the fullerene site all implied that the ASU should contain two halves of the fullerene molecule with occupancies of 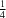, rotated relative to each other through a dyad perpendicular to the fourfold axis. Since building a dis­ordered fullerene model and fitting it into the electron-density map in such a complicated case, it is much easier to handle an entire C_60_ molecule rather than its halves; thus, two complete C_60_ molecules with 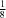 occupancy were modelled into the electron-density map.

For a similar reason, the structure solution and initial refinement were carried out in the space group *I*4, despite the fact that already during data processing and scaling the possibility of higher symmetry, *I*422, was quite obvious. However, after a preliminary analysis of the electron-density map, which strongly indicated a model of two dis­ordered fullerene molecules on the fourfold axis, it was decided that the first stages of structural analysis would be carried out with the assumption of lower symmetry, where atoms of the two whole fullerene molecules would have an occupancy of 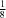, rather than 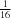, as required by the higher *I*422 symmetry.

After numerous attempts to model the dis­ordered fullerene molecules, it became obvious that the only possible mode of their arrangement requires exact alignment of the molecular twofold axes with the crystallographic fourfold axis of this site. During the first cycles of refinement of the complete model with two properly oriented fullerene molecules, it turned out that they tend to spin out of alignment of their twofold axes with the fourfold axis of the space group, that the *R* factor is still high (∼23%) and that the model becomes inconsistent with the electron-density map because the positions of the fullerene atoms no longer match the electron-density peaks. To proceed with the refinement it was, therefore, necessary to solve the problem of fullerene rotation in the electron-density map. *SHELXL* is probably the only program that has a mechanism to prevent such unwanted rotations and at the same time to correctly refine the structure. The twofold axes of the fullerene molecule (there are 15 different twofold axes passing through opposite C—C bonds) that are of interest in this context run through the centres of two opposite 6–6 bonds, *i.e.* the common bonds of two six-membered rings. To keep the fullerene in a proper orientation, first the centres of two selected bonds have to be fixed on the fourfold axis. To achieve this, the SUMP instruction of the *SHELXL* program was used. SUMP allows free variables to be logically related and is most commonly used to restrain the occupancy factors of more than two atoms sharing the same site, or three or more alternative conformations. SUMP can do much more but is rarely used for anything else. This command has one very important and unique feature, *i.e.* it makes it possible to use custom restraints by defining a linear equation describing a relationship between selected free variables along with a standard deviation that determines the weight of such a restraint. These variables are then refined under the control of the defined equations. The general command syntax is:

SUMP *c sigma c1 m1 c2 m2* …, where *c* = *c1* * *fv(m1)* + *c2* * *fv(m2)* + …,

*fv(m1), fv(m2)*, … are refinable free variables, and *sigma* determines the weight of the restraint.

In the present case, to fix the centres of bonds on the fourfold axis located at (*x* = 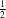, *y* = 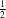), the SUMP command with the following parameters was used (the example is for one selected bond, *i.e.* C5—C6):

SUMP 1.0 0.01 1.0 2 1.0 3,

where *c, c1, c2* = 1.0, *fv(m1)* and *fv(m2)* with *m* values of 2 and 3 correspond to the second and third free variables, assigned with starting values of the *x* coordinates of atoms C5 and C6, respectively.

Analogously, for the *y* coordinates of atoms C5 and C6, the SUMP command is:

SUMP 1.0 0.01 1.0 4 1.0 5, 

where the values 4 and 5 refer to the free variables defined as the *y* coordinates of atoms C5 and C6, respectively. To carry out the refinement, the atom instructions for C5 and C6 in the *SHELXL* command file should be as follows:

C5 1 21.000000 41.000000 61.000000 10.12500 0.05

C6 1 31.000000 51.000000 61.000000 10.12500 0.05.

This means that the coordinates *x1, y1* and *x2, y2* of the C5 and C6 atoms, respectively, will be refined while complying with the following equations: 

1 * *x1* + 1 * *x2* = 1 and 1 * *y1* + 1 * *y2* = 1,

*i.e.* with *c, c1, c2* = 1, *x1* = *fv*(2), *x2* = *fv*(3), *y1* = *fv*(4) and *y2* = *fv*(5). The *z* coordinate of both atoms, defined as free variable 6 [*fv*(6)] will be the same for both atoms. The initial coordinates of the C5 and C6 atoms are given using the FVAR instruction defining the refined variables:

scale 21 31 41 51 61

FVAR 2.76192 0.47145 0.52856 0.51238 0.48761 0.63740

After applying the appropriate SUMP, FVAR and atom instruction (for two selected bonds per each fullerene molecule), the refinement became stable and converged with *R* ∼ 15%. Despite the complex dis­order, the positions of the fullerene C atoms fit the electron density well and the molecular twofold axes of the fullerene models coincided with the crystallographic fourfold axis.

However, a detailed analysis of the symmetry of the model suggested that the true symmetry of the crystal may be higher than assumed during data processing and initial refinement, and that the correct crystal class is 422. Accordingly, further refinement was carried out in the space group *I*422, which allowed model reduction by one-half by reason of the twofold symmetry perpendicular to the principal fourfold axis. The new ASU contains a model consisting of only 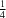 of a resor­cin­arene moiety decorated with a lower-rim alkyl chain and an upper-rim l-phenyl­alanine arm (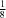 of the whole capsule) and one fullerene molecule with an occupancy of 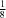 (although we know that according to the 422 symmetry, the ASU should contain only 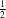 of the C_60_ molecule with an occupancy of 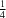). In the following refinement, after each round of minimization, the *COOT* program was used for visualization of the electron-density maps and for manual rebuilding of the atomic models.

Since the refinement was stable at this stage, it was possible to remove the redundant half of the symmetrically dependent C_60_ molecule. Ultimately, the structure was refined in the very rare space group *I*422 with a model consisting of only 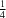 of a resorcinarene molecule with one lower-rim alkyl chain and one l-phenyl­alanine arm (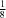 of the whole capsule) at full occupancy, and of 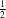 of the fullerene molecule sitting on the fourfold axis, with an occupancy of 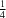. To perform the refinement with only 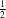 of the C_60_ molecule (*i.e.* with a model that is correctly represented in the ASU from a crystallographic point of view), it was necessary to change the method of restraining the direction of the twofold axis of the fullerene molecule. The SUMP instruction cannot be used for this purpose any longer because there is no possibility to define the centre of the 6–6 bond, as one of the atoms forming this bond is not part of the model (it is generated by the fourfold symmetry). An alternative method to achieve a similar effect as with the SUMP instructions is based on the observation that in the proper fullerene orientation certain groups of its atoms must have the same *z* coordinate, which can be refined as one free variable. After updating the *SHELXL* instruction file and defining all *z* coordinates of the fullerene molecule by reference to FVAR free variables, the refinement was still stable. Because the fullerene molecule shows a high degree of dis­order, with four alternative orientations of the molecule with an occupancy of 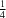, during refinement, geometric restraints were used for all the C_60_ bond lengths and angles, using the DFIX and DANG instructions with default weights. The definition of such restraints was relatively straightforward, because the C_60_ fullerene molecule has only two different bond angles with ideal values of 108° and 120° and two types of C—C bonds, *i.e.* double (6–6) and single (6–5), with average bond lengths of 1.397 and 1.454 Å, respectively (Schein & Sands-Kidner, 2008 ▸; Aoyagi *et al.*, 2014 ▸). The stability of the dis­ordered l-phenyl­alanine residues and alkyl groups during refinement was enforced by appropriate stereochemical restraints with default weights.

Introducing into the model double conformations of the l-phenyl­alanine ring and alkyl groups, which are clearly visible in the electron-density map, coupled with anisotropic ADP refinement, reduced the *R* factor to ∼9%.

### 2.5. Sample preparation for NMR measurements in the solid state   

65 mg of (**3**)_2_ 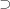
C_60_ was ground in an agate mortar, mixed with 42 mg of ground lead nitrate and packed into a 4 mm zirconia rotor in order to prepare the sample for solid-state NMR measurements. The ^207^Pb resonance of lead nitrate was used as an internal NMR thermometer (Beckmann & Dybowski, 2000 ▸). The ^13^C longitudinal relaxation times of fullerene C_60_ in (**3**)_2_ were determined in the temperature range 184–333 K at a magnetic field of 11.73 T using a Bruker AVANCE II 500 MHz machine. The direct carbon-detected inversion-recovery method was used. Typical parameters were as follows: 8–24 scans for each of the 8–14 incremented delays, spectral widths of 400 ppm, acquisition times of 160 ms and 90° pulses of 3.5 µs. The recycle delay was always longer than five times the *T*_1_ of the ^13^C nuclei of C_60_. A Bruker DVT 4 mm MAS probe head was used. In the temperature range 248–333 K, measurements were performed under magic angle spinning conditions with a frequency of 2–3 kHz, while below 248 K the sample was not spun in order to avoid large temperature gradients. Similar experiments were performed at a magnetic field of 7.04 T using a Bruker AVANCE II 300 MHz spectrometer equipped with a Bruker VTN 4 mm probe head. The temperature range was 194–298 K. Since samples in the VTN probe head are much less prone to temperature gradients, we used magic angle spinning over the whole temperature range.

### 2.6. Theory used for calculation of dynamic parameters in the solid state from NMR data   

In isotropic liquids, where molecules undergo rotational Brownian motion, the nuclear spin relaxation processes are caused by time-dependent interactions involving the nuclear spins. For spin-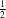 nuclei, such as ^13^C (spin *S*), the most significant relaxation mechanisms often involve dipole–dipole (DD) interactions with neighbouring protons (spins *I*) and the anisotropic part of the shielding tensor (chemical shift anisotropy, CSA). The commonly used expression for the longitudinal relaxation rate constant reads (Canet, 1998 ▸): 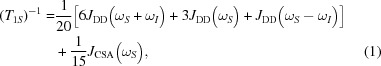
where *J*_DD_(ω) and *J*_CSA_(ω) are dipolar and CSA spectral densities, respectively, taken at frequency ω. For rigid spherical-top molecules, these quantities are given by 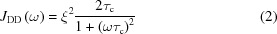
and 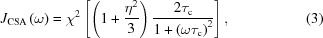
with 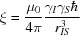
and 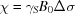 being interaction strengths for the DD and CSA relaxation mechanisms, respectively. γ denotes the nuclear magnetogyric ratio, *B*_0_ the induction of the external magnetic field, *r_IS_* the internuclear distance and τ_c_ the rotational correlation time. Δσ and η are called anisotropy and asymmetry of the shielding tensor. Both these quantities can be expressed in terms of the principal components σ*_ii_* of the traceless part of the shielding anisotropy tensor: 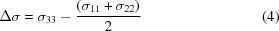


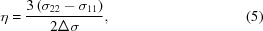
with |σ_22_| ≤ |σ_11_| ≤ |σ_33_|. Equation (2) describes the dipole–dipole relaxation in a rotating rigid body. It is not directly applicable to the case of the ^13^C nuclei in C_60_ relaxed by remote protons of the hosting capsule because the rotational diffusion of C_60_ leads to modulation of not only the direction of the internuclear vector but the length of the latter as well. Equations taking into account such modulations are available (Bernatowicz *et al.*, 2006 ▸), but they were derived for systems involved in discrete internal motions of molecular fragments, with the latter dynamics being described by an exchange matrix. Such a matrix for C_60_ tumbling in (**3**)_2_ is unknown, so that even the advanced and flexible formalism of Bernatowicz *et al.* (2006 ▸) fails to describe the dipole–dipole mechanism for this system. Inspection of equation (2) reveals, however, some general features of the dipole–dipole mechanism which are of relevance to this work: (i) the contribution from the dipole–dipole mechanism decreases with the sixth power of the internuclear distance, so that even a large number of remote protons may not overwhelm the relaxation of the ^13^C nuclei in fullerene if they are far enough away and (ii) for motion fast enough (the so-called extreme narrowing regime, where ωτ*_c_* ≪ 1), the contribution of the DD mechanism, in contrast to the CSA mechanism, is independent of induction of an external magnetic field. These two properties of the dipole–dipole relaxation process render it separable from that due to the field-dependent CSA mechanism. The latter seems to be faithfully described in model (**3**)_2_ 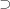
C_60_ by equation (3). The CSA mechanism should prevail over that of DD in appropriately high magnetic fields (due to its dependence on the squared strength of the external magnetic field). Thus, relaxation measurements performed at two (or more) suitably different magnetic fields enable evaluation of the contribution of the CSA mechanism with good accuracy. In order to exploit equation (3) for evaluation of the correlation time (τ_c_) of the rotational tumbling of C_60_, knowledge of the symmetric part of CSA in fullerene is required. The full shielding tensor was determined for solid fullerene (Yannoni *et al.*, 1991 ▸) to be σ = (40, 186, 220) ppm, which corresponds to Δσ = −163.0 ppm and η = 0.31.

### 2.7. Differential scanning calorimetry (DSC) measurements   

The phase transitions and thermal stability of the samples were studied using DSC. The DSC data were obtained using a Q200 scanning calorimeter (TA Instruments). Samples were placed in aluminium *T*_zero_ hermetic pans. An empty pan was used as a reference. Data analysis was carried out using the TA Universal Analysis application. Three cycles of cooling, heating and cooling at a rate of 10 K min^−1^ for each scan in the temperature range from 90 to 355 K were carried out in a flow of nitro­gen.

## 3. Results and discussion   

The X-ray crystal structure of (**3**)_2_ 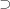
C_60_ at 100 K was determined using synchrotron data. Despite the initially high *R* factor (∼24% for the isotropic model without dis­order), inspection of the electron-density map revealed that the fullerene molecule was clearly visible with pronounced, fairly well separated, peaks (Fig. 2 ▸ *a*). Therefore, we undertook a tedious refinement procedure (described in detail in the *Experimental* section) involving the following crucial steps: (i) initial refinement of the structure in the low-symmetry space group *I*4 with a molecule of **3** located at the fourfold axis, noticing a mismatch of symmetry between the crystallographic fourfold symmetry and the *C*_2_ molecular symmetry of C_60_; (ii) location and modelling of two complete C_60_ molecules with 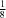 occupancy into the electron-density map at the position where the molecular twofold axis aligns with the crystallographic fourfold axis of this site; (iii) imposing of restrictions onto fullerene rotation during refinement; (iv) refinement in the higher-symmetry group *I*422. Finally, the refinement con­verged at *R* ≈ 9%. The ASU contains 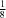 of the (**3**)_2_ 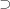
C_60_ complex. The host capsule (**3**)_2_ resides at the site with 422 symmetry. The encapsulated C_60_ molecule also resides at this site, but since it has only *C*_2_ molecular symmetry in the [001] direction of its orientation, it generates a symmetry mismatch at this site, leading to discrete dis­order. This implies that the remaining symmetry operations at this 422 site (90° rotation about the fourfold axis and rotations about the diagonal twofold axis) generate C_60_ copies in different orientations with respect to the host molecule (Figs. 2 ▸ *b*, 2 ▸ *c* and 2 ▸ *d*). There are eight halves of the fullerene in the entire cell, which form four complete molecules. They constitute two pairs, rotated relative to each other around the twofold axis of C_60_ (coinciding with the fourfold axis of the *I*422 space group) by an angle of 42.3°. The fullerene molecules forming a given pair are rotated by 90° relative to each other as a result of the action of the fourfold symmetry. The second pair is generated by twofold rotation perpendicular to the crystallographic *c* axis. However, considering the molecular 422 symmetry of the (**3**)_2_ host, in fact, all possible arrangements are identical from a chemical point of view.

**Figure 3:**
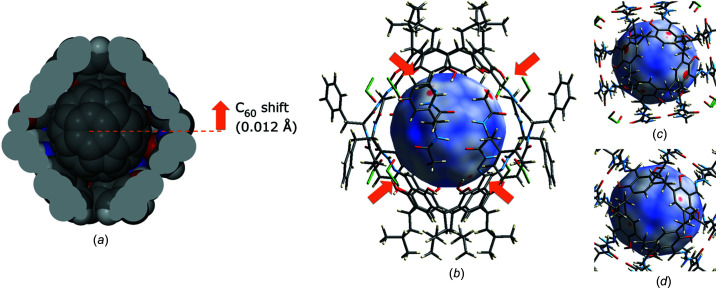
Analysis of the intermolecular interactions. (*a*) van der Waals representation of C_60_ inside the cavity of (**3**)_2_ [(**3**)_2_ was sliced in order to visualize the interior of the cavity]. (*b*)–(*d*) Analysis of the intermolecular contacts by mapping of the intermolecular distances onto an C_60_ isosurface generated at 0.002 au electron density (as implemented in *CrystalExplorer*; Turner *et al.*, 2017 ▸), with (**3**)_2_ shown in stick representation and C_60_ as the isosurface; the surface is colour-coded for host–guest contacts after subtraction of the sum of the van der Waals radii (red < 0, white = 0 and blue > 0). (*b*) A side view, (*c*) a 90° view of the upper hemisphere and (*d*) a 90° view of the bottom hemisphere.

A detailed analysis of the molecular geometry and intermolecular contacts was carried out in *CrystalExplorer* (Fig. 3 ▸). The intramolecular distances were mapped on the molecular surface of C_60_ (0.002 au electron-density isosurface). The C_60_ guest is located close to the central part of the cavity but not at its geometric centre. The shift of C_60_ from the geometric centre of the cavity is 0.012 Å in the direction of one of the resorcin[4]arene cups (Fig. 3 ▸ *a*). It is important to note that the off-centre shift of C_60_ results in different intermolecular interactions with the two hemispheres of the capsule that are clearly reflected in the maps. One (upper) hemisphere exhibits close contacts of the hydrazone C atoms of (**3**)_2_ to C_60_ (Figs. 3 ▸ *b*, 3 ▸ *c* and 3 ▸ *d*); the closest distances are for C⋯C interactions of 3.15 and 3.21 Å, and the distances to the centroids of the resorcinol rings are longer. For the second (lower) hemisphere, the contacts are longer; the distances for C⋯C interactions are 3.40 and 3.46 Å, and the distances to the centroids of the resorcinol rings are similar.

Before considering the symmetry mismatch in the X-ray crystal structure of (**3**)_2_ 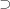
C_60_, the position and dynamics of the C_60_ molecule were not clear. However, after the successful resolution of the crystal structure it can be concluded that at 100 K the molecule of C_60_ is statistically but not thermally dis­ordered over discrete symmetry-equivalent positions. An analysis of the geometry indicates that, despite rather weak intramolecular interactions, the thermal motion of C_60_ is frozen. In order to check the dynamics of C_60_ over a wider temperature range, we used solid-state ^13^C NMR. The ^13^C longitudinal relaxation times of fullerene C_60_ in complex (**3**)_2_ 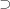
C_60_ were determined in the temperature range 184–333 K at two magnetic field strengths, *i.e.* 7.04 and 11.73 T. Although the theory for the calculation of thermal-motion parameters from these NMR data is well elaborated, in this case, the analysis proved to be more complex than initially anticipated. It was complicated by the time-dependent changes ongoing in the solid state and biased by the rather unexpected interactions with atmospheric oxygen or water. Below, we present a detailed analysis of these problems which, after appropriate compensation, allowed us to obtain correct dynamic parameters for C_60_.

**Figure 4:**
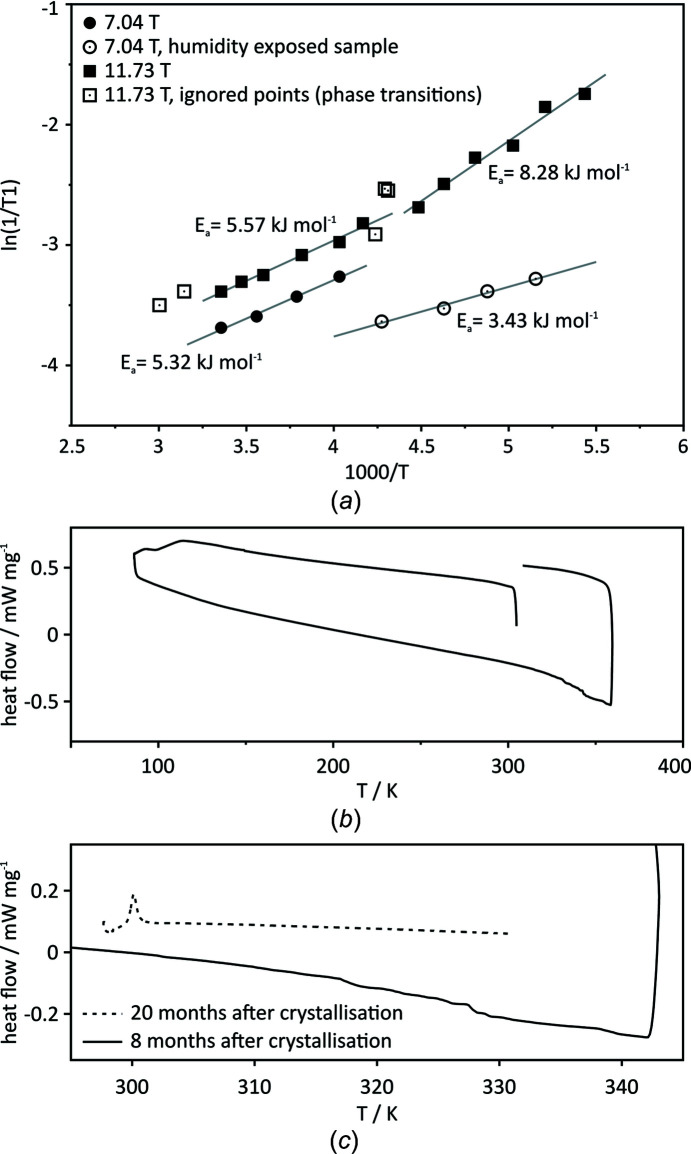
(*a*) Arrhenius plots of the relaxation data for the ^13^C NMR signal of C_60_ in (**3**)_2_ 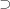
C_60_ (7.04 and 11.73 T). (*b*) Differential scanning calorimetry (DSC) curve for sample (**3**)_2_ 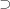
C_60_, measured eight months after crystallization. (*c*) Comparison of a fragment of the DSC curves for sample (**3**)_2_ 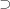
C_60_ measured at eight and 20 months after crystallization.

The sample for NMR measurements was obtained by vacuum drying of (**3**)_2_ 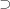
C_60_ (amorphous solid obtained after synthesis), which was subsequently ground, mixed with ground lead nitrate, packed into a zirconia NMR rotor and hermetically sealed. The Arrhenius plot of the relaxation data in the range 298–184 K at 11.73 T is shown in Fig. 4 ▸(*a*) (solid squares). The data reveal non-linearity over the whole temperature range, indicating that the thermal motion of C_60_ has different activation energies under different temperature regimes. The data were fitted with two lines: one with an activation energy of *E*_a_ = 5.57 ± 0.30 kJ mol^−1^ in the temperature range 298–240 K and the other with an activation energy of *E*_a_ = 8.28 ± 0.65 kJ mol^−1^ in the temperature range 223–184 K. The data for the intermediate temperature range (236–232 K; open squares in Fig. 4 ▸ *a*) and above 298 K depart significantly from both of these trends, likely indicating phase transitions. In order to detect these phase transitions, the DSC method was employed, which indeed confirmed the high-temperature phase transition (above 298 K; Figs. 4 ▸ *b* and 4 ▸ *c*). However, no phase transition was detected at lower temperatures. In order to validate the above NMR data, we remeasured the sample using a different machine (7.04 T), which should eliminate incident sample-independent errors and enable separation of different relaxation mechanisms. The Arrhenius fit in the temperature range 298–248 K delivers *E*_a_ = 5.32 ± 0.31 kJ mol^−1^, which is consistent with the data measured at 11.73 T (solid circles in Fig. 4 ▸ *a*). However, the data below 248 K (open circles in Fig. 4 ▸) are distinctly different from this fit and also from the data measured previously at 11.73 T. The Arrhenius fit in this range yields *E*_a_ = 3.43 ± 0.29 kJ mol^−1^. It should be noted here that although the experiments were carried out for the same sample, they were not recorded at the same time; during the time lapse between the experiments, the sample spent a few months in contact with humid air. Although the chemical formula did not change during this time (as confirmed by solution NMR), apparently some structural changes in the solid sample did occur which were ultimately reflected in the fullerene rotational mobility and, in consequence, in the rates of its spin-relaxation processes. The DSC profiles recorded for the same sample before and after ‘aging’ (of 12 months) also reveal structural changes and substantial differences in the characteristics of the phase transition. The phase transition for the sample before aging is diffused between 298 and 333 K, while after aging a very sharp peak at ∼300 K was found (see Fig. 4 ▸ *c*).

**Figure 5:**
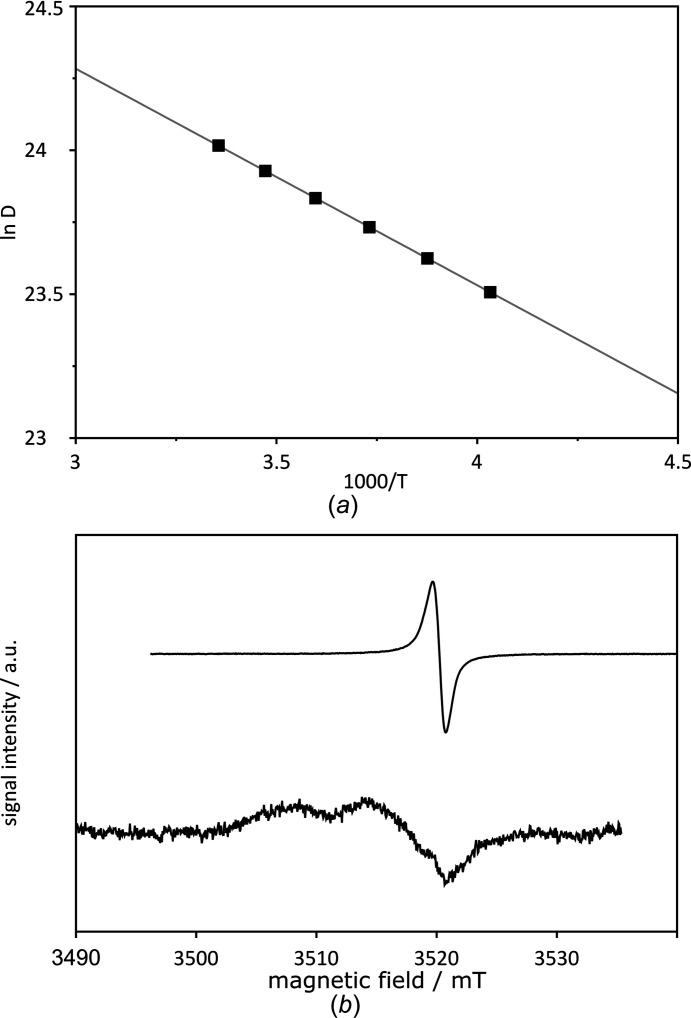
(*a*) Arrhenius plot of the rotational diffusion coefficient, *D*, of C_60_ in (**3**)_2_ 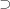
C_60_ obtained from the CSA relaxation data listed in Table 2 ▸. The activation energy is *E*_a_ = 6.25 kJ mol^−1^, while the pre-exponential factor was *D*_0_ = 3.36 × 10^11^ s^−1^. (*b*) EPR spectrum of the ‘off-the-shelf’ C_60_ (upper panel; material exploited in the synthesis of the complex) and EPR spectrum of complex (**3**)_2_ 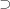
C_60_ (lower panel).

A similar slope of the Arrhenius fits in the range 248–298 K for data measured at different magnetic fields enables separation of the contributions from different spin-relaxation mechanisms within this temperature range (Canet, 1998 ▸). Specifically, we can exploit the fact that within the so-called extreme narrowing regime, the CSA contribution to spin relaxation is field dependent, while other contributions (comprising the DD mechanism to other nuclei and/or the DD mechanism to unpaired electrons) are not [see equations (2) and (3)]. The obtained contributions are listed in Table 2 ▸. Based on these contributions and using the components of the known fullerene CSA tensor (Yannoni *et al.*, 1991 ▸), the application of Equation (3) enables a straightforward determination of the rotational correlation times, τ_c_, of fullerene and, in consequence, its rotational diffusion coefficient *D* = (6τ_c_)^−1^. The latter quantities are also listed in Table 2 ▸. The Arrhenius plot of *D* is shown in Fig. 5 ▸(*a*). The activation energy obtained from this plot is *E*_a_ = 6.25 kJ mol^−1^, while the pre-exponential factor is *D*_0_ = 3.36 × 10^11^ s^−1^. Our results for C_60_ tumbling inside the supramolecular capsule (**3**)_2_ agree surprisingly well with similar data determined *via* NMR spectroscopy (Johnson *et al.*, 1992 ▸) and QENS (Quasi Elastic Neutron Scattering) (Neumann *et al.*, 1991 ▸) for neat C_60_. We performed investigations similar to Johnson *et al.* (1992 ▸), *i.e.* a multiple-field NMR relaxation study, in order to separate spin relaxation induced by CSA from those induced by other mechanisms. They obtained *E*_a_ = 5.85 ± 0.42 kJ mol^−1^, *D*_0_ = 2.06 × 10^11^ s^−1^ and *D* = 1.8 × 10^10^ s^−1^ at 283 K, while the QENS technique delivered a slightly lower *E*_a_ = 3.38 ± 1.45 kJ mol^−1^ and *D* = 1.4 × 10^10^ s^−1^ at 260 K (Neumann *et al.*, 1991 ▸). More recently, very careful investigations of C_60_ dynamics by NMR spectroscopy in the solid state were performed by Izotov & Tarasov (2002 ▸).

In conclusion, it appears that the activation energy for reorientations of free and encaged C_60_ in the solid state is very similar. Also, the rotational diffusion coefficients seem to be very similar or even slightly higher for the encaged fullerene as compared to the free molecule. Table 2 ▸ also reveals that CSA is not the main relaxation mechanism for ^13^C spins of C_60_ fullerene in (**3**)_2_ 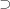
C_60_, even in a magnetic field of 11.7 T. This points to a surprisingly high contribution of non-CSA mechanisms, taking into account that for neat fullerene at 11.7 T only about 10% of its relaxation comes from mechanisms other than CSA [see Fig. 2 ▸ in Johnson *et al.* (1992 ▸)]. A possible explanation for this large amount of non-CSA relaxation usually involves internuclear dipole–dipole interactions. However, the X-ray crystal structure of (**3**)_2_ 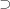
C_60_ reveals that there are no protons approaching the encaged C_60_ molecule at distances shorter than ∼2.95 Å, which would correspond to ^13^C–^1^H dipole–dipole coupling of only 1.2 kHz. Such couplings have a negligible impact on the ^13^C spin relaxation in C_60_. An alternative explanation for the high contribution of non-CSA mechanisms to relaxation may come from dipole–dipole interaction with unpaired electrons. We acquired Electron Paramagnetic Resonance (EPR) spectra of the C_60_ sample used in the synthesis (off-the-shelf sample) and of (**3**)_2_ 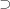
C_60_ (Fig. 5 ▸ *b*). The EPR spectrum of ‘off-the-shelf’ C_60_ shows pronounced EPR effects. It has been demonstrated previously that although C_60_ itself is not paramagnetic, its derivatives obtained by reactions with molecular oxygen from the air are (Paul *et al.*, 2002 ▸). We assume that this effect is also reflected in the spectrum of the ‘off-the-shelf’ C_60_ sample. The EPR effects for (**3**)_2_ 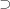
C_60_ are much smaller but certainly not negligible. Electronic paramagnetism detected in the sample can be responsible for the observed high non-CSA contributions to relaxation. It should be noted that in Johnson *et al.* (1992 ▸), which describes ^13^C relaxation experiments on neat C_60_, similar problems with intensive paramagnetic relaxation were not reported. However, in the cited study, in the course of the sample preparation, C_60_ was extracted with supercritical CO_2_ and the obtained material was sealed under vacuum, so that the sample was protected against the impact of oxygen.

## 4. Conclusions and outlook   

We have described the synthesis of a molecular container formed by zipping together two resorcin[4]arene hemispheres *via* a system of circular β-sheet hydrogen bonds between their peptidic arms. The container, with a largely hydro­philic inner surface, has been loaded with C_60_ fullerene and crystallized. The crystal structure studied at 100 K using synchrotron radiation reveals that the complex occupies the 422 site of the space group *I*422, which is compatible with the molecular symmetry of the container but not of the C_60_ molecule, leading to its discrete dis­order. Resolving a fourfold dis­order of a fullerene molecule in the electron-density maps is quite a challenge, but it could be completed successfully, as confirmed by the final electron-density maps, and the convergent and stable least-squares refinement carried out using the advanced features available in the *SHELXL* program. We hope that our success and example will encourage other crystallographers to attack similar problems which at first glance appear to be intractable. The crystallographic study has been supplemented by solid-state NMR experiments over the temperature range 184–333 K, carried out for both fresh and aged samples, and using spectrometers with different magnetic field strengths (7.04 and 11.73 T). It appears that the activation energy for reorientations of free and encaged C_60_ in the solid state is very similar. Also, the rotational diffusion coefficients seem to be very similar or even slightly higher for the encaged fullerene compared to the free one. The NMR results also show time-dependent phase transitions in the sample, confirmed at higher temperature by DSC experiments that also affect the activation energy for reorientations. A detailed study of this phase transition and an X-ray crystal structure determination of the high-temperature phase are the subject of an ongoing project.

## Supplementary Material

Crystal structure: contains datablock(s) I. DOI: 10.1107/S2052520620009944/lo5072sup1.cif

Structure factors: contains datablock(s) I. DOI: 10.1107/S2052520620009944/lo5072Isup2.hkl

CCDC reference: 1987205

## Figures and Tables

**Table 1:** Data collection and refinement statistics for the (**3**)_2_ 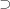
C_60_ structure

**Data collection**	
Radiation source	P13 EMBL, Petra III, Hamburg
Wavelength (Å)	0.8266
Temperature (K)	100
Space group	*I*422
Cell dimensions *a*, *c* (Å)	21.910, 25.44
Resolution range (Å)	16.60–0.76 (0.81–0.76)†
Number of reflections	5405
Completeness native (%)	92.5 (55.0)†
Redundancy	5.75 (1.82)†
〈*I*/σ*I*〉	34.6 (8.42)†
*R*_merge_ (%)	4.9 (7.7)†
Wilson *B*-factor (Å)^2^	4.87
	
**Refinement**	
Refinement program	*SHELXL* (Sheldrick, 2015*b* ▸)
Resolution (Å)	16.60–0.76
No. of reflections	5405
*R* (%)	8.89
No. of atoms in ASU	40.5
〈*B*〉 (Å)^2^	5.24

† Values in parentheses correspond to the last resolution shell.

**Table 2:** Contributions of various relaxation mechanisms to spin-relaxation rates *R*1 = *T*1^−1^ of ^13^C nuclei in C_60_ at 7.04 and 11.73 T, and rotational diffusion coefficients, *D* = (6τ_c_)^−1^, determined from CSA relaxation rates as a function of temperature

*T* (K)	*R*1_CSA_ at 7.04 T (10^−2^ s^−1^)	*R*1_CSA_ at 11.73 T (10^−2^ s^−1^)	*R*1_other_ (10^−2^ s^−1^)	*D* (10^10^ s^−1^)
298	0.51	1.40	1.96	2.69
288	0.55	1.53	2.10	2.47
278	0.61	1.68	2.27	2.24
267	0.67	1.85	2.46	2.03
258	0.75	2.07	2.69	1.82
248	0.84	2.32	2.96	1.62
